# Bioaccessible (Poly)phenols of Winery Byproducts Modulate Pathogenic Mediators of Intestinal Bowel Disease: *In Vitro* Evidence

**DOI:** 10.1021/acs.jafc.5c00916

**Published:** 2025-04-23

**Authors:** Vicente Agulló, Cristina García-Viguera, Sonia Medina, Raúl Domínguez-Perles

**Affiliations:** † Departamento de Tecnología Agroalimentaria, EPSO, Universidad Miguel Hernández, Carretera Beniel km 3.2, 03312 Orihuela, Alicante, Spain; ‡ Laboratorio de Fitoquímica y Alimentos Saludables (LabFAS), CEBAS, CSIC, Campus Universitario de Espinardo, Edificio 25, 30100 Murcia, Spain

**Keywords:** enological byproducts, bioaccessibility, phenolic compounds, intestinal bowel disease, oxidative stress, in vitro models

## Abstract

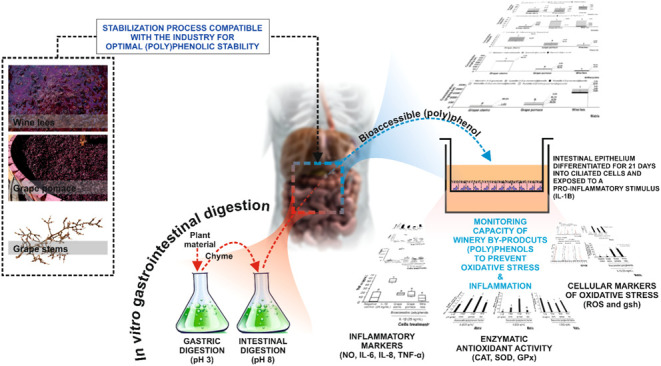

Intestinal inflammation entails a multifactorial pathophysiology, frequently treated by using anti-inflammatory drugs with severe side effects. At the same time, bioactive compounds present in plant materials and derived residues could contribute to reducing the use of such medications in terms of dosage and treatment length. Thus, the phytochemicals of winery byproducts, mainly represented by (poly)phenols, display significant anti-inflammatory and antioxidant potential. However, the functionality of bioaccessible fractions remains underexplored. This study uncovers the capacity of bioaccessible (poly)phenols of winery byproducts to modulate inflammatory mediators and secondary oxidative stress (OS). After *in vitro* simulated digestion, bioaccessible (poly)phenols exhibited significant inhibitory capacity of nitric oxide, interleukin (IL)-6, IL-8, and TNF-α production and prevented OS, lowering reactive oxygen species (ROS) resulting from disturbed cell metabolism while preserving the molecular machinery of cells, involving glutathione, catalase, superoxide dismutase, and glutathione peroxidase. The results retrieved suggested the relevance of specific profiles for efficiently preventing inflammation.

## 1 Introduction

Inflammatory bowel disease (IBD) is a chronic disorder characterized by a lack of gastrointestinal tissue homeostasis and the development of an autoreactive immune response. In this regard, the histopathology associated with this process reveals the infiltration of immune cells into the lamina propria, triggered by proinflammatory interleukins secreted by epithelial cells. Moreover, the enhanced cell metabolism resulting from inflammation augments the production of reactive oxygen species (ROS) responsible for oxidative stress (OS) and the lack of permeability of the mucosa, which speed up the inflammatory process.^1^

Nowadays, the treatment of this pathology is based on the use of anti-inflammatory drugs, immunosuppressive antibodies, and receptor inhibitors to modulate the immune response.^2^ However, these treatments entail detrimental side effects that could jeopardize the appropriate clinical evolution of patients.

In the sought preventive agents that reduce the dose of anti-inflammatory drugs, dietary (poly)phenols have been stressed due to their capacity to modulate the molecular mechanism ongoing in IBD with minimal side effects. Indeed, these phytochemicals downregulate the synthesis and secretion of proinflammatory and chemotactic interleukins and enzymes, limiting the proliferation and infiltration of immune cells responsible for autoreactive damaging responses and enhancing the cells’ antioxidant defense.^3^ In this connection, (poly)phenols are the most characteristic phytochemicals in winery byproducts (grape stems, grape pomace, and wine lees) that have been described as sustainable sources of proanthocyanidins and catechin derivatives, phenolic acids, stilbenes, flavonols, and anthocyanins.^4^ These (poly)phenolic profiles have led to envisaging alternative uses of enological residues toward health-protecting coproducts.^5^

Despite this functional potential, evidence gathered on the anti-inflammatory capacity of winery byproducts’ (poly)phenols accounts for a serious constraint since most related research has been focused on the composition of fresh materials, whose concentrations and quantitative profiles rarely remain after digestion. To overcome this limitation, new assessments of the bioactivity of the bioaccessible fractions are required.^6^ This is key because, although gastrointestinal digestion allows extracting the bioactive components of food through enzymatic, chemical, and mechanical events, all these factors, together with the interactions of (poly)phenols with other food components, could interfere with their release and stability, which condition the final bioactivity.^6−8^ For that, the assessment of the behavior of bioaccessible fractions in complex biological systems will allow demonstrating their real interest as food sources of functional compounds and make it possible to valorize residues and thus avoid their negative impact on the sustainability and competitiveness of the industries,^9−11^ as recommended by the European authorities.^12^

Based on the above, the present article uncovers the capacity of bioaccessible (poly)phenols of wine byproducts to modulate the profile of inflammatory mediators involved in the course of IBD, namely, nitric oxide (NO) and proinflammatory interleukins (IL-6, IL-8, and TNF-α), resorting to an *in vitro* model of IBD. Beyond this, indicators of secondary OS and redox balance of intestinal cells (reactive oxygen species (ROS), glutathione (GSH), catalase (CAT), superoxide dismutase (SOD), and glutathione peroxidase (GPx)) were monitored to shed light on the capacity of bioaccessible (poly)phenols to prevent secondary OS and enhance the tissue damage that make up the course of IBD.

## 2 Materials and Methods

### 2.1 Chemicals and Reagents

Standard compounds catechin, 1,4-chlorogenic acid, resveratrol, quercetin 3-*O*-glucoside, and cyanidin 3-*O*-glucoside were obtained from Sigma-Aldrich (Steinheim, Germany). Enzyme-linked immunoSorbent assay (ELISA) kits for the detection and quantification of IL-6, IL-8, TNF-α, ROS, and GSH, as well as for the determination of CAT, SOD, and GPx activity, were purchased from Abcam (Cambridge, UK), along with [1-(4-chloromercuryphenyl-azo-2-naphtol)] (Mercury Orange) and 2′,7′-dichlorofluorescein diacetate (DCFDA). Formic acid was obtained from Panreac (Castellar del Vallés, Barcelona, Spain). All LC–MS-grade solvents were purchased from JT Baker (Phillipsburg, NJ). Ultrapure water was produced by using a Millipore water purification system.

Trypsin–EDTA, Eagle’s minimum essential medium (EMEM), l-glutamine, fetal bovine serum (FBS), penicillin/streptomycin, and essential amino acids were purchased from Gibco (Thermo Fisher Scientific, Madrid, Spain). Flat-bottomed 24-well plates were purchased from Corning (New York, NY).

### 2.2 Plant Material Collection and Processing

Solid (grape stems and pomace) and semisolid (wine lees) winery byproducts were obtained from the ecological Monastrell grape (*Vitis vinífera* L. var. Monastrell) under the Protected Designation of Origin (Bodegas Via Elena S.L., Jumilla, Murcia, Spain). These byproducts resulted from an alcoholic fermentation carried out with destemmed grapes and indigenous yeasts. The byproducts were dehydrated by applying a time–temperature gradient (initial temperature (75 °C)–final temperature (60 °C)) for 10 h until constant weight, according to the procedure described recently in the literature.^13^ In this work, the dehydration conditions were developed to preserve phenolic compounds from thermal degradation while ensuring the microbial safety of the stabilized materials obtained.^13^ The safety of the dehydrated byproducts was confirmed through microbiological assays, which demonstrated compliance with European sectoral regulations (Commission Regulation (EC) No. 2073/2005). Dried materials were ground to a fine powder, stored in a desiccator, and protected from light for further application of simulated *in vitro* gastrointestinal digestions.

### 2.3 Simulated *In Vitro* Gastrointestinal Digestions

Simulated gastrointestinal digestions were performed on dehydrated winery byproducts’ powder following the INFOGEST methodology,^14,15^ with minor modifications.^16^ Briefly, gastric digestion was simulated by mixing 500 mg of the samples with 15 mL of a simulated gastric fluid (SGF) stock electrolyte solution (Supporting Table 1). Pepsin (EC 3.4.23.1) was dissolved in SGF. Digestions were performed under continuous agitation at 52 oscillations per minute for 2 h at 37 °C. The final pH was adjusted to pH 3 by adding 1 M HCl. The reactions were ended by adding a 0.2 M sodium hydroxide solution. To simulate the intestinal phase of digestion, a simulated intestinal fluid (SIF) was prepared (Supporting Table 1). Intestinal enzymes pancreatin (EC 232-468-9) and pancreatic lipase (EC 3.1.1.3) were dissolved in SIF. Additionally, frozen porcine bile salts were added to reach a final concentration of 10 mM. The pH of the SIF was adjusted to 8.0 using 1 M NaOH. Gastrointestinal digestion was completed by performing the intestinal stage for 2 h at 37 °C. The enzymatic reactions were stopped by immediately freezing, at −80 °C, the bioaccessible fractions. For assessing the bioaccessible (poly)phenolic content, using HPLC–PAD-ESI-MSn, samples were centrifuged at 2000 rpm for 5 min at 4 °C and filtered through a 0.22 μm PVDF filter (Millipore, MA).

### 2.4 HPLC–DAD-ESI-MSn Analysis of Phenolic Compounds

The bioaccessible extracts obtained according to the procedure described in the previous subsection were directly analyzed by LC–MS without further extractions. The chromatographic separation and mass spectrometry analysis of the (poly)phenols present in the bioaccessible fractions was performed following the methodology described in the literature.^4^ Briefly, phenolic compounds were analyzed by HPLC–PDA-ESI/MS^n^ using a Luna C18 column (250.0 × 4.6 mm, 5.0 μm, Phenomenex, Macclesfield, UK) and an Agilent 1100 series HPLC system equipped with a diode array and mass detector (Agilent Technologies, Waldbronn, Germany). Chromatographic separation was performed with a mobile phase of deionized water/formic acid (99:1, v/v) (solvent A) and acetonitrile/formic acid (99:1, v/v) (solvent B). To resolve analyte separation, a flow rate of 1 mL/min was applied upon the linear gradient scheme (t in min; %B): (0; 5%), (15; 15%), (30; 30%), (40; 50%), (45; 95%), and (50; 5%). The equipment consisted of a binary pump (model G1312A), an autosampler (model G1313A), a degasser (model G1322A), a photodiode array detector (model G1315B), and an ion trap spectrometer (model G2445A) equipped with an electrospray ionization interface and controlled by LCMSD software (v. 4.1 Agilent Technologies) operating according to the chromatographic and ionization specifications described by Costa-Pérez et al.^4^ The quantification was done on chromatograms recorded at 280 nm for proanthocyanidins and catechin derivatives, 330 nm for phenolic acids and stilbenes, 360 nm for flavonols, and 520 nm for anthocyanins, with calibration curves freshly prepared each day of analysis.

### 2.5 Cell Line, Culture Conditions, and Assessment of Modulators of Intestinal Inflammation

The colorectal Caco-2 (ATCCHTB37) human cell line was obtained from the American Type Culture Collection (ATCC, Rockville, MD) (passage number between 16 and 18). For this, when confluent, Caco-2 cells were allowed to differentiate for 21 days before the experiments to express phenotypic characteristics of the intestinal epithelium, according to the descriptions available in the literature. Cell monolayer integrity was checked by reference to the transepithelial electrical resistance (TEER) measured by a Millicell ERS (Millipore Co., Bedford, MA) using Ag–AgCl electrodes, according to the manufacturer’s instructions.^17^ Once differentiated, the culture media was replaced by fresh growth media supplemented with the bioaccessible fraction of winery byproducts’ (poly)phenols, previously filtrated through a 0.22 μm PVDF filter (Millipore, MA) at a 1:10 (v/v) ratio that exhibited no cytotoxicity (data not shown). After 1 h of exposure, 25 ng/mL (final concentration) of IL-1β was added to all wells (except negative controls) and maintained for 10 h, when the highest level of immune modulatory IL was secreted.^18^ Afterward, the cells were detached with trypsin-–EDTA at 0.05% and centrifuged at 5000 rpm for 10 min, and the cells and supernatants were kept at −80 °C. The supernatants were assessed for the contents of IL-6, IL-8, and TNFα, following the manufacturer’s instructions. The quantification limits of the ELISA kits for IL-6, IL-8, and TNFα were 0.81, 12.30, and 4.32 pg/mL, respectively.

### 2.6 Determination of the Enzymatic Antioxidant Activity and Oxidative Stress Markers in Caco-2 Cells

The cells collected after the treatments specified as described in the previous subsection were assessed concerning the SOD, CAT, and GPx enzymatic antioxidant activity with specific ELISA kits according to the manufacturer’s instructions (Abcam, Cambridge, UK). Beyond the enzymatic antioxidant activity, variations of the OS markers (ROS and GSH) were determined by resorting to two-color flow cytometry.

The analysis of oxidative stress markers was developed by two-color flow cytometry assays used to assess oxidative stress markers, namely, glutathione (GSH) and reactive oxygen species (ROS). Flow cytometry analyses were conducted on a BD FACSCalibur cytometer (Becton Dickinson, CA), and 5000 gated events were collected from each sample. Data analysis was performed using Winlist, version 9.0 (Verity Software Hose, Inc., Topsham, ME).

For cytometric determinations, after harvesting Caco-2 cells cultured and treated as described in Section 2.5, they were resuspended in 100 μL of PBS and stained separately for GSH and ROS with [1-(4-chloromercuryphenyl-azo-2-naphtol)] (Mercury Orange) (Abcam, Cambridge, UK) and 2′,7′-dichlorofluorescein diacetate (DCFDA), respectively, following the manufacturer’s instructions and according to the flow cytometry settings described in the literature.^19,20^ Briefly, for cytometric determinations, Caco-2 cells (3.0 × 10^5^ cells/mL) were cultured in six-well culture plates. After 24 h, culture media was removed, and cells were exposed to FBS-free culture media supplemented with bioaccessible (poly)phenols of grape stems, grape pomace, and wine lees at a ratio of 1:10 (v/v). After 1 h of exposure, 25 ng/mL (final concentration) IL-1β was added to all wells (except negative controls) and maintained for 10 h. Afterward, cells were detached with trypsin–EDTA at 0.05% and centrifuged at 5000 rpm for 10 min. The supernatants were removed, and the cells were resuspended in 100 μL of PBS, stained separately for the aforementioned oxidative stress markers, and analyzed by flow cytometry.

For monitoring the GSH level, cells were stained with [1-(4-chloromercuryphenyl-azo-2-naphtol)] (Mercury Orange), following the procedure described previously.^21^ Briefly, the cells were incubated with 40 μM Mercury Orange for 5 min, at RT, in the dark. Then, the cells were washed, resuspended in 100 μL of PBS, and kept on ice until acquisition by a BD FACSCalibur cytometer (Becton Dickinson, CA). To set up the intracellular concentration of ROS, cells were incubated with 20 μM 2′,7′-dichlorofluorescein diacetate (DCFDA) solution at 37 °C for 45 min and protected from light. After a final wash, cells were resuspended in 100 μL of PBS and kept on ice until acquisition by the BD FACSCalibur cytometer (Becton Dickinson, CA).

### 2.7 Statistical Analysis

All experimental conditions were performed in sextuplicate (*n* = 6), and the data were expressed as the mean ± standard deviation (SD). According to the normal distribution and homogeneity of variance of the data (determined by Shapiro–Wilk (<50 samples) and Levene tests, correspondingly), the obtained results were subjected to a one-way analysis of variance (ANOVA), and when statistical differences were identified, the variables were compared using Tukey’s multiple range test. Significant differences were set at *p* < 0.05.

Relationships between the concentration of bioactive (poly)phenols and variations in the level of inflammation and OS markers and mediators were analyzed by Spearman’s correlation analysis, and significant correlations were set at *p* < 0.05.

All statistical analyses were performed with the SPSS program, version 25.0 (SPSS Inc., Chicago, IL).

## 3 Results and Discussion

According to evidence of a heterogeneous (poly)phenolic composition in winemaking byproducts recently described by our research team,^4,13^ the phenolic burden of enological residues includes a large diversity of phenolic compounds, being this variability associated with a range of biological functions (*e.g*., anti-inflammatory activity or OS prevention). These bioactivities result from interaction with molecular mediators of transversal processes involved in multiple pathophysiological situations,^22^ including the diverse clinical phenotypes of IBD.^23^ In this concern, the thermal gradient applied with stabilization purposes preserved, to the highest extent, the quantitative (poly)phenolic profile of winery byproducts while guaranteeing the microbial safety of the resulting materials.^13^ Nonetheless, for the scientific sound implementation of valorization processes based on the (poly)phenolic content and its functional consequences, central questions need to be answered: To what extent does the winery byproducts’ (poly)phenolic profile change after digestion? Does the remaining profile constitute an operative concentration to prevent inflammation and the associated OS? Thus, the bioaccessible fractions of grape stems and pomace and wine lees need to be assessed for their (poly)phenolic profile and anti-inflammatory power to shed light on these questions.

### 3.1 Bioaccessibility of Separate Winery Byproducts’ Phenolic Compounds

**Figure 1:**
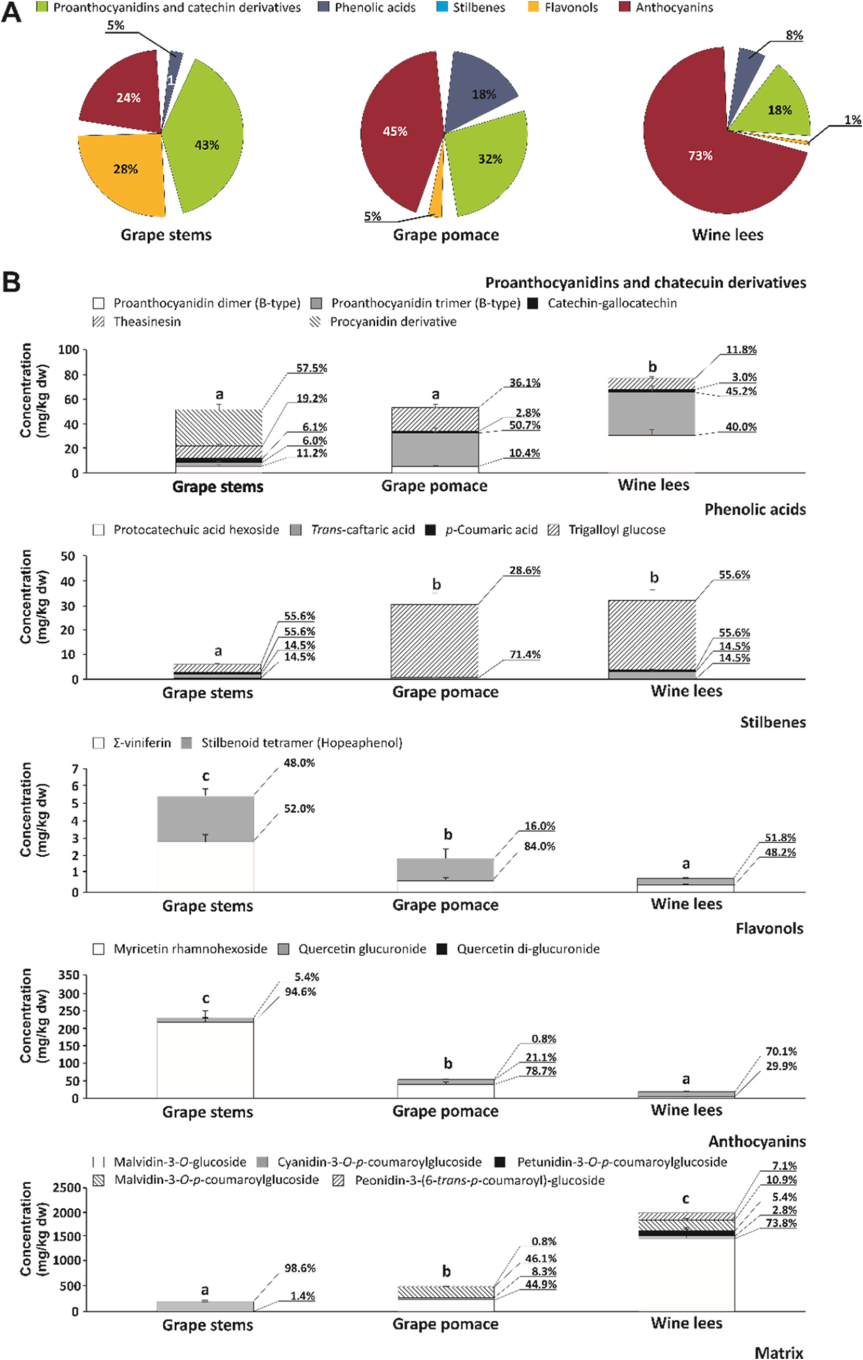
Percentage of phenolic classes in the digestion products of the separate winery byproducts (A) and content (mg/kg dw) of total bioaccessible proanthocyanidins and catechin derivatives, phenolic acids, stilbenes, flavonols, and anthocyanins, with an indication of the percentage of contribution of each phenolic (B). Values are calculated as the mean ± SD (*n* = 6). Bars with distinct lowercase letter classes differ significantly according to one-way analysis of variance (ANOVA) and Tukey’s multiple range test at *p < 0.05.

The bioaccessibility analysis of (poly)phenols present in winery byproducts exhibited the different contributions of the separate phenolic classes in grape stems (485.91 mg of total (poly)phenols/kg dw) relative to grape pomace and wine lees (642.02 and 2132.69 mg of total (poly)phenols/kg dw, respectively) (Figure 1A). Thus, while the main contribution to bioaccessible (poly)phenols in grape stems was provided by proanthocyanidins and catechin derivatives (43.0%), followed by flavonols and anthocyanins (28 and 24%, respectively), anthocyanins were the most abundant type in grape pomace and wine lees, accounting for 45.0 and 73.0%, respectively. In grape pomace and wine lees, proanthocyanidins and catechin derivatives were also found in significant concentrations, contributing by providing 32.0 and 18.0% of the total phenolic burden, respectively. In the three materials, phenolic acids provided a lower supply, ranging between 5 and 18% (Figure 1A).

The preponderance of flavonols and anthocyanins in the bioaccessible fraction agrees with previous descriptions of the enhanced stability of flavonoids during digestion.^24^ These phenolics should be considered critical for the biological scope of a given plant material upon oral administration. Indeed, these results show the relevance of sorting out the (poly)phenols’ bioaccessibility according to the stability under digestive physicochemical conditions conferred by the diverse chemical structure since the obtained profiles differ significantly from those described in fresh materials.^25^

When focusing on the quantitative profile corresponding to each separate class concerning proanthocyanidins and catechin derivatives, the total bioaccessible content was significantly higher in wine lees (76.65 mg/kg dw) than in grape stems and pomace (51.39 and 52.99 mg/kg dw, respectively), which displayed a 33.3% lower content, on average. This result allows hypothesizing a protective effect of the overall physicochemical features and composition of wine lees on (poly)phenols, which could constitute a differentiation fact compared with grape stems and pomace.

When analyzing individual proanthocyanidins and catechin derivatives, it was found that, in grape stems, they were mainly made up according to the following decreasing order of concentration: nonidentified procyanidin derivative (57.5%) > theasinesin (19.2%) > procyanidin dimer (B-type) (11.2%) > procyanidin trimer (B-type) and catechin-gallocatechin (both at 6.1%, on average). On the other hand, after digestion of grape pomace and wine lees, proanthocyanidin trimer (B-type) (26.87 and 34.68 mg/kg dw, respectively) was the most contributing compound to the proanthocyanidin and catechin derivative class, followed by theasinensin and proanthocyanidin dimer (B-type) (19.14 and 30.66 mg/kg dw) in grape pomace and wine lees, correspondingly (Figure 1B).

Concerning total phenolic acids, the highest bioaccessibility corresponded to grape pomace and wine lees, which exhibited no significantly different concentration (31.07 mg/kg dw, on average) and surpassed the content of grape stems by almost 81.0% (*p* < 0.05) (Figure 1B). Concerning the contribution of separate individual phenolic acids, the concentration of trigalloyl glucose (54.5–98.1%) was the predominant in all byproducts. In addition, *trans*-caftaric acid was also a relevant contributor to the phenolic acids’ burden of grape stems (25.3%) and wine lees (8.4%) (Figure 1B).

Despite the low concentration of stilbenoids in the bioaccessible fraction of the three winery byproducts under consideration, some of them were at a level higher than the limit of quantification of the analytical technique. The highest concentration corresponded to grape stems (5.35 mg/kg of dry weight (dw)), followed by grape pomace (1.88 mg/kg of dry weight) and wine lees (0.75 mg/kg of dry weight) (Figure 1B), which was calculated as the sum of concentrations of two individual compounds, ∑-viniferin and hopeaphenol (stilbenoid tetramer). Both contributed similarly to the total stilbene burden in all of the matrices (Figure 1B).

Total bioaccessible flavonols were obtained from grape stems, grape pomace, and wine lees and were the highest in grape stems (225.01 mg/kg dw), representing concentrations 77.0 and 91.7% greater than those recorded in grape pomace and wine lees, respectively (Figure 1B). In grape stems and grape pomace, the most abundant flavonol found was myricetin rhamnohexoside (212.89 and 40.68 mg/kg dw, respectively), while in wine lees, quercetin glucuronide was the main contributor (Figure 1B).

Finally, anthocyanins were found at the highest concentration in wine lees (2004.76 mg/kg dw), surpassing the amounts found in grape stems and grape pomace by 90.5 and 74.8%, respectively (Figure 1B). The preponderant anthocyanin in the bioaccessible fraction of grape pomace and wine lees was malvidin 3-*O*-glucoside (226.58 and 1479.78 mg/kg dw, respectively) and malvidin 3-*O*-*p*-coumaroylglucoside (232.73 and 218.91 mg/kg dw, respectively) (Figure 1B).

In summary, findings reported in the present article on the quantitative (poly)phenolic profile of winery byproducts support a significant (poly)phenolic decrease compared with previous reports on the phenolic burden of these plant materials (fresh and stabilized materials), as expected. In this concern, the (poly)phenolic degradation described in the present work as a result of gastrointestinal digestion constitutes an accepted trend for several vegetable matrices, which share most phenolic compounds with winery byproducts,^6,26^ as well as with residues derived from other plant-food production processes.^27^ In the present work, it was observed that the modification of the quantitative (poly)phenolic profile did not occur to the same extent across all byproducts, which displayed significant bioaccessibility differences. Nonetheless, this fact allows management alternatives to take advantage of such differences for practical applications. In this concern, the different profiles generated in each byproduct during gastrointestinal digestion led to envisaging new formulations based on the combination of three residues, each of them supplying bioactive compounds with specific functionals, thus contributing to additive mixes or synergies between the different materials.^4^ This is critical because most functional studies are performed on (poly)phenolic extracts obtained from fresh materials, which leads to overestimating the real biological power.^28^ In this frame, this effect seems to be a significant modulator of the biological potential associated with winery byproducts’ (poly)phenols,^29^ which should not be ignored to shed light on the actual biological scope and the development of feasible valorization procedures.^9^

In this context, the dependency of (poly)phenols’ bioaccessibility on external conditions, beyond their chemical traits (including sterified or glycosylated forms of phenolics and complexation with macromolecules) (e.g., proteins, carbohydrates like starches or dietary fiber, lipids, etc.), deserves to be considered as a factor involved in the stability of (poly)phenols during digestion or release of phenolic compounds not available as free compounds in the plant material.^30^ Indeed, the (poly)phenols’ stability during digestion is influenced by the chemical characteristics of the separate compounds, the physical properties of the plant material, and the physicochemical and enzymatic conditions of the digestion process; together, these factors contribute to changes in the quantitative phenolic profile,^31^ as evidenced by the higher bioaccessibility of wine lees (poly)phenols recorded in the present work. However, further characterization is needed to understand the extent to which these changes would influence the potential of diverse materials to serve as dietary sources of healthy bioactive compounds,^32,33^ whose final effect is significantly associated with the bioaccessibility of their (poly)phenolic profiles.^34,35^ The result of the digestion impact in the (poly)phenols present in the intestinal lumen, available for cell uptake, has to be comprehensively characterized, beyond their phytochemical profile, on bioactivity (*e.g*., regarding anti-inflammatory and OS prevention power),^32^ to further support the interest of a given matrix as a functional product.

### 3.2 Modulating Inflammatory Markers by Bioaccessible (Poly)phenols of Winery Byproducts

Previous studies have described changes in the intestinal epithelium during chronic inflammatory diseases (*e.g*., IBD). Simultaneously, due to these histopathological changes, the damaged epithelium triggers an autoreactive immune response and inflammatory process by secreting a range of interleukins, establishing a hallmark of the dialog between epithelial and immune cells.^36^

Interleukins are low-molecular-weight peptides and glycoproteins that act as immunological mediators to maintain cell homeostasis.^36^ Under specific proinflammatory stimuli, interleukins activate protecting reactions,^37^ which may disturb tissue integrity and barrier functions and, finally, under abnormal intensity and reactivity features of the response, could define pathological phenotypes.^38^

**Figure 2:**
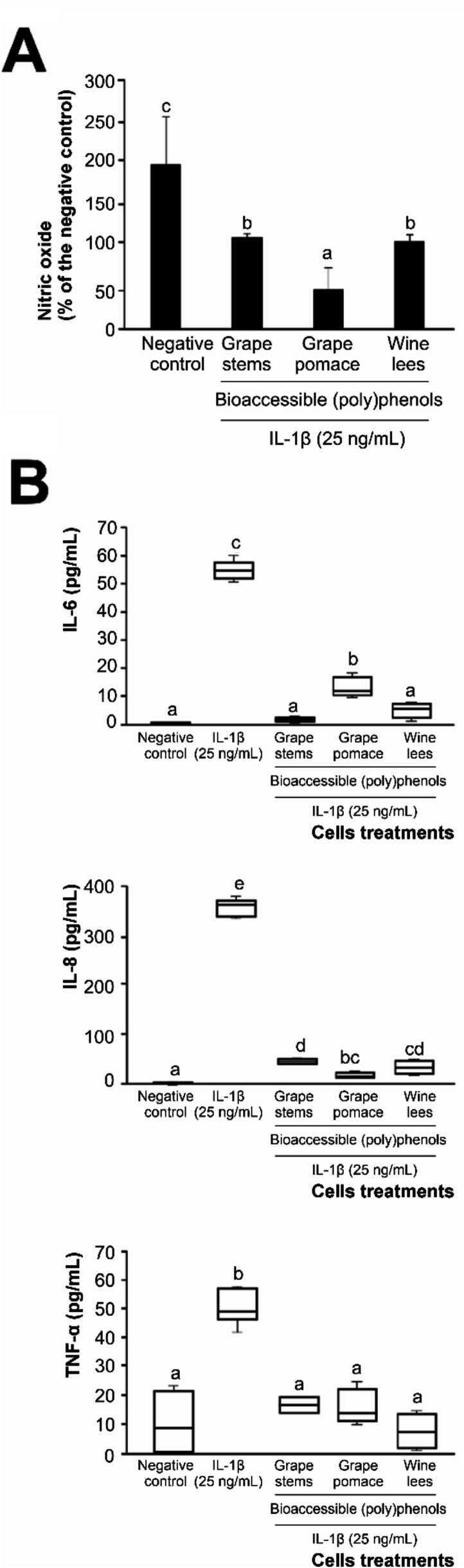
Capacity of the bioaccessible (poly)phenolic fractions of winery byproducts (grape stems, grape pomace, and wine lees) to modulate the inflammatory mediators: nitric oxide (A) and interleukins (IL)-6, IL-8, and TNF-α (B), secreted by Caco-2 cells exposed to an inflammatory stimulus (25 ng/mL IL-1β). Distinct lowercase letters indicate significantly different values at *p* < 0.01 according to one-way analyses of variance (ANOVA) and Tukey’s multiple range test (*n* = 6).

To date, the pharmacological approach to this pathological situation involves administering antibodies or receptor inhibitors that modulate the immune response.^2^ Nonetheless, in recent years, promising and less aggressive strategies have emerged for fine-tuning the interleukin profile, focusing on the administration of dietary bioactive compounds as personalized preventive measures for individuals predisposed to inflammatory and immune-mediated conditions.^39^ To gather evidence about the extent to which bioaccessible (poly)phenols of winery byproducts modulate the interleukin profile under proinflammatory conditions at the intestinal location, human colorectal (Caco-2) cells were exposed to IL-1β (25 ng/mL), thus triggering the inflammatory phenotype.^40^ Changes in the concentration of key proinflammatory mediators were monitored, including NO and interleukins IL-6, IL-8, and TNFα, after supplementation of (poly)phenolic extracts of digestion products (Figure 2A,B). However, before that, the potential cytotoxic effect of the bioaccessible (poly)phenolic fraction on Caco-2 cell line viability was assessed. No evidence was obtained concerning the cytotoxicity of the bioaccessible (poly)phenolic fractions of winery byproducts (data not shown).

The application of digestion products reduced the NO production by 55.9%, on average, compare to the epithelial cells exposed only to the proinflammatory stimulus (positive control); notably, (poly)phenols from stressed grape pomace lowered the production of NO to the highest extent (by 76.7%) (Figure 2A). This result indicated an effective anti-inflammatory capacity.^4,20^ Given the roles of NO in signaling and immunomodulation, decreasing its secretion would help prevent the molecular cascade responsible for the onset of the inflammatory response.^41^ Accordingly, the anti-inflammatory capacity of bioaccessible (poly)phenols of winery byproducts was further explored by analyzing changes in the production of proinflammatory cytokines IL-6, IL-8, and TNF-α (Figure 2B).

The exposition of Caco-2 cells to IL-1β (inflammatory stimulus) at 25 ng/mL induced augmentation of IL-6 (56.7 pg/mL), the broadly described regulator of inflammation and immune response. The proinflammatory activity of this interleukin is mediated by the differentiation of lamina propria-resident macrophages into migratory antigen-presenting cells, which are involved in triggering the acquired immune response.^40^ Interestingly, the bioaccessible fractions of grape stems, grape pomace, and wine lees significantly decreased IL-6 production by Caco-2 cells under proinflammatory conditions by 89.2%, on average, until 1.1–12.8 pg/mL, almost the level recorded in the untreated negative control cells (Figure 2B).

In the context of inflammation, complementary to IL-6, IL-8 functions as a powerful chemotactic factor for leukocytes upon the induction of integrin secretion. Both IL-6 and IL-8 facilitate enhanced adhesion of immune cells to the endothelium and derives the activation of diverse cell types toward effector phenotypes.^41,42^ Consequently, decreasing IL-8 secretion would contribute to the resolution of inflammation. In this study, (poly)phenols present in the bioaccessible fractions of all three enological residues reduced IL-8 expression by 90.9%, on average (Figure 2B), highlighting their valuable contribution to the prevention and resolution of inflammation.

Concerning the secretion of TNF-α by the intestinal epithelial cells under proinflammatory conditions, an average value of 51.3 pg/mL was recorded. Interestingly, the bioaccessible (poly)phenols of all winery byproducts almost restored the TNF-α concentration to that observed in untreated cells (negative control) (14.1 pg/mL, on average) (Figure 2B), which is in good agreement with their powerful anti-inflammatory capacity. The relevance of this result lies in the proinflammatory character of TNF-α, which is responsible for a wide range of signaling events within cells that polarize the cell death pathway toward necrosis, thus contributing to perpetuating the inflammatory status.^43^

According to the main results obtained, the bioaccessible fraction presented a high capability to prevent inflammation by modulating the signaling molecules responsible, to a high extent, for the differentiation and activation of immune-competent cells. Moreover, interestingly, this efficiency is higher than that of the (poly)phenolic fraction of fresh residues reported in previous studies.^13^ These results advise about the applicability of these underused materials for the design and development of further treatments targeting intestinal inflammation.^4,20^

### 3.3 Modulation of Oxidative Stress by Bioaccessible (Poly)phenols of Winery Byproducts

**Figure 3:**
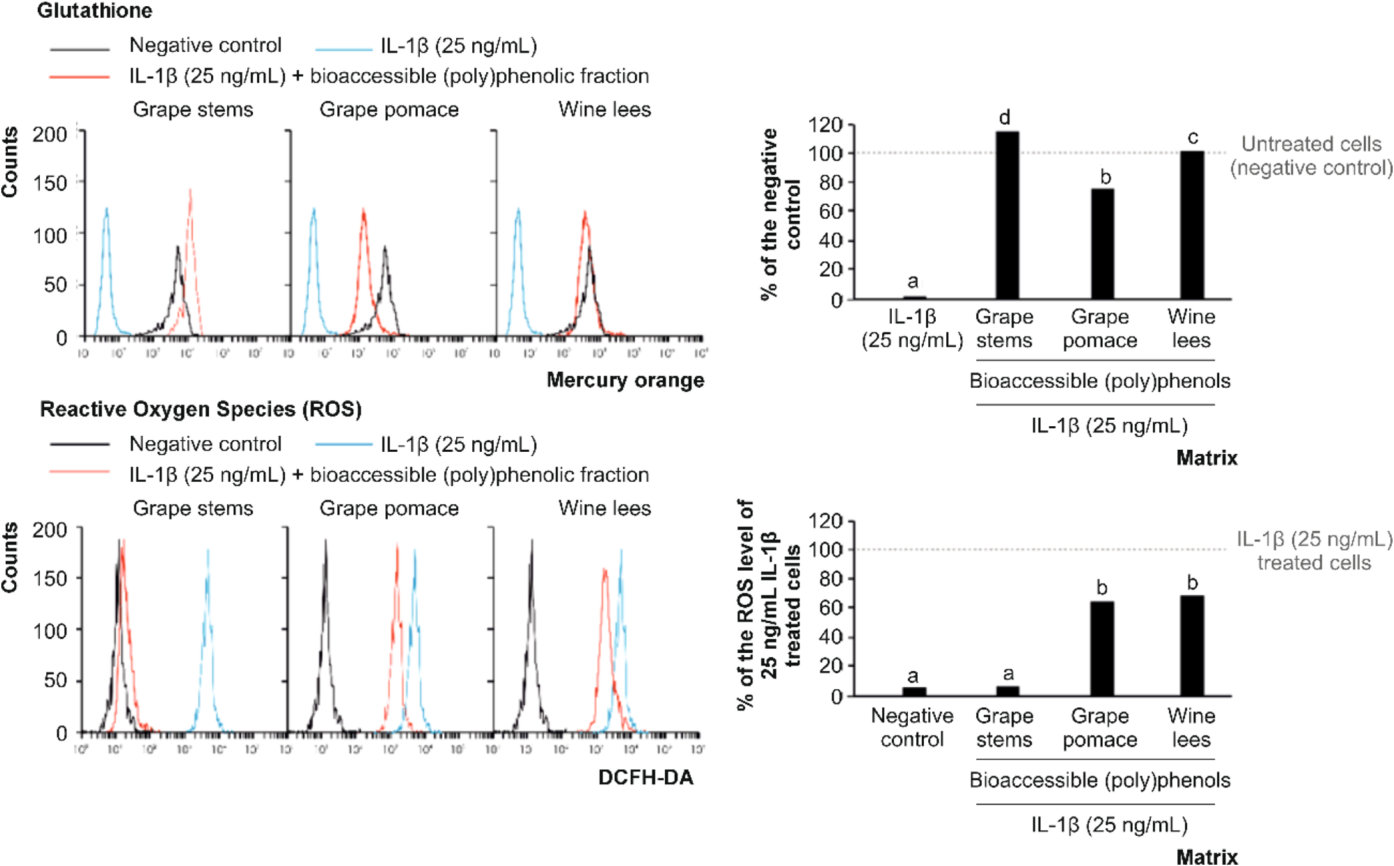
Histograms of representative Mercury Orange-based glutathione (GSH) and DCFH-DA-based reactive oxygen species (ROS) of Caco-2 cells exposed to an inflammatory stimulus (25 ng/mL IL-1β) alone or in combination with the bioaccessible (poly)phenolic fraction derived from grape stems, grape pomace, and wine lees and the quantitative analysis of GSH and ROS relative to the negative and positive controls, respectively. Different lowercase letters indicate significantly different values at *p* < 0.01 according to one-way analyses of variance (ANOVA) and Tukey’s multiple range test (*n* = 6).

**Figure 4:**
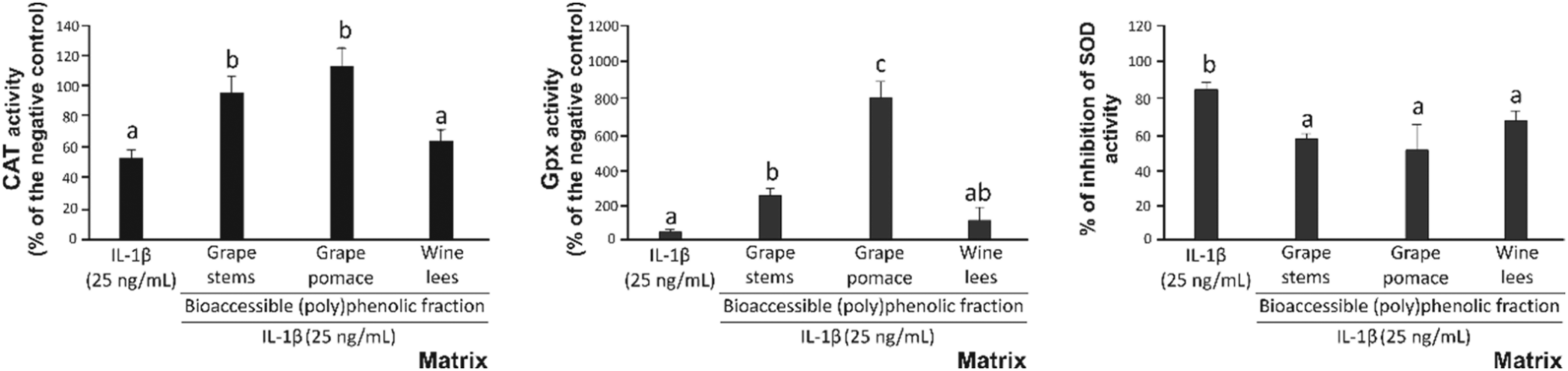
Capacity of the bioaccessible (poly)phenolic fraction derived from grape stems, grape pomace, and wine lees to restore the enzymatic antioxidant (catalase (CAT), glutathione peroxidase (GPx), and superoxide dismutase (SOD)) activity in Caco-2 cells exposed to an inflammatory stimulus (25 ng/mL IL-1β). Different lowercase letters indicate significantly different values at *p* < 0.01 according to one-way analyses of variance (ANOVA) and Tukey’s multiple range test (*n* = 6).

In the absence of harmful environments, ROS are maintained at nontoxic levels by the molecular machinery of cells.^44^ However, specific conditions (*e.g*., proinflammatory or pro-oxidant environments) augment their production, contributing to the progression of various diseases; consequently, elevated ROS levels can have damaging effects on critical cell molecules (*e.g*., lipid peroxidation or alteration of the nucleotide sequence in the DNA). This situation demands alternative treatments aiming at reducing their concentration to guarantee cell survival and ensure the proper functioning of organs and systems.^45,46^ Moreover, identifying interventions with operative antioxidant effects (beyond the anti-inflammatory capacity) would support the development of valorization alternatives for these materials. This fact prompted us to monitor OS and the antioxidant status of intestine epithelial cells under inflammatory conditions by a two-step characterization; first, monitoring the intracellular levels of GSH and ROS (Figure 3) and, second, assessing bioaccessible (poly)phenols for their capacity to protect the enzymatic antioxidant machinery of cells in the form of CAT, SOD, and GPx (Figure 4).

#### 3.3.1 Glutathione and Reactive Oxygen Species in the Caco-2 Intestinal Monolayer Epithelium

The evaluation of winery byproducts’ capability to prevent the consumption of intracellular GSH indicated the contribution of bioaccessible compounds to the protection of the intestinal epithelium against inflammation. In this concern, (poly)phenols derived from grape stems, grape pomace, and wine lees, as released into the intestinal lumen from gastrointestinal digestion, exhibited a significant capacity to maintain the concentration of GSH compared with the level recorded in cells after exposure to proinflammatory IL-1β. This result indicated the contribution of (poly)phenols of enological residues to defense cells against OS secondary to inflammation (Figure 3). When comparing the efficiency of separate matrices, the highest activity found corresponded to grape stems and wine lees (10.8-fold higher concentrations than the control cells, on average), while bioaccessible (poly)phenols from grape pomace only maintained 72.3% of the GSH concentration (Figure 3). Therefore, the bioaccessible fractions of enological residues preserved GSH and thus contributed to preventing OS^47^ since GSH, according to its reducing properties, acts as the most important scavenger of electrophilic and oxidant species generated due to cell metabolism while acting as a cofactor of antioxidant enzymes, protecting against mutagenic effects and lipid peroxidation.^48^

Among the bioaccessible (poly)phenols from winery byproducts, those derived from grape stems demonstrated the highest capacity to significantly lower the ROS level in cells exposed to inflammatory conditions. This reduced ROS levels to those observed in untreated cells (negative control) (Figure 3). Also, although less efficient, the digestion products from grape pomace and wine lees lowered the ROS levels by 34.4%, on average (Figure 3).

According to these results, it was found that the bioaccessible fractions assessed protected the antioxidant machinery of cells, possibly due to the collaborative antioxidant activity of individual phenolics released into the intestinal lumen during digestion. Moreover, these fractions were more efficient in protecting cells than the hydromethanolic extracts obtained from fresh and dehydrated materials.^13^ This demonstrates the capacity to control the side effects of inflammation, tentatively associated with the close relationship between the phenolic profile and the capacity to maintain the redox balance;^49^ therefore, the applicability of winery byproducts for developing future treatments gains a better prognosis for intestinal inflammation.^50^

#### 3.3.2 Modulation of Catalase, Superoxide Dismutase, and Glutathione Peroxidase Antioxidant Enzymatic Activity

In addition to quenching molecules responsible for lowering the concentration of harmful free radicals, the antioxidant defense grid of cells is also integrated by antioxidant enzymes. This machinery includes SOD, CAT, and GPx.^51^

When analyzing the capacity of bioaccessible (poly)phenolic fractions to maintain the activity of CAT in cells exposed to proinflammatory IL-1β, it was observed that only the bioaccessible fraction of grape stems and grape pomace recovered the CAT activity of untreated cells (Figure 4). Similarly, analysis of GPx activity indicated that the bioaccessible fractions from all residues augmented its concentration by 5.8-fold, 18.9-fold, and 5.6-fold for grape stems, grape pomace, and wine lees, respectively, compared to the values recorded in cells exposed to IL-1β (Figure 4).

Finally, given the role of SOD in cell defense mechanisms against OS,^51^ identifying molecules or natural extracts that help to maintain its activity during inflammation would complement therapeutic approaches for diseases characterized by enhanced OS. In this context, the bioaccessible fractions concerning winery byproducts assessed restored significantly the SOD activity between 18.5 and 35.4% (Figure 4).

This ability to preserve the activity of antioxidant enzymes constitutes the first defense line against oxidative insults and plays a central role in protecting living organisms against inflammation,^52^ thus contributing to improving the prognosis of IBD patients.

Despite the promising *in vitro* findings, to date, it is important to acknowledge that extrapolation of these benefits to humans remains limited due to the complexity of *in vivo* settings of gastrointestinal pathophysiology and interindividual variability. To overcome this limitation, future studies should explore, *in vivo*, the actual bioavailability, including absorption, distribution, metabolism, and excretion of the (poly)phenolic burden of winery byproducts, which is influenced by the chemical traits of the bioactive compounds and their capacity to reach the different tissues and cell types and interact with the molecular pathways involved in cellular uptake and signaling. All these factors are closely influenced by dosage, which, in turn, is modulated by interindividual metabolic variability. However, to date, limited research has been conducted to establish standardized dosages for these compounds across different population groups (*e.g*., men, women, adults, senior, etc.), which will require additional research actions in the medium term to fine-tune the recommended dietary allowance (RDA) for such bioactive compounds, ensuring safety while avoiding potentially harmful interactions.

Another factor that could influence the biological interest (efficacy or toxicity) of dietary (poly)phenols administered to living beings is their interaction with other bioactive components in the food matrix. In this regard, despite (poly)phenols being featured by a valuable anti-inflammatory affects through antioxidant activity, enzyme inhibition, and modulation of gene expression, their combination with polyunsaturated fatty acids can enhance the functional scope. These molecular interactions could modify the bioavailability, individual metabolism, and, thereby, the eicosanoid profiles and signaling pathways affected by their reactivity against inflammation and oxidative stress triggers and mediators. Moreover, (poly)phenols’ interaction with other bioactive compounds and macromolecules, as well as with gut microbiota, would play a pivotal role in modulating inflammatory processes.^53^

### 3.4 Correlation Analyses

The identification of individual (poly)phenols responsible for preventing inflammation and OS was set up by applying Spearman’s correlations between the bioaccessible (poly)phenols of winery byproducts and the level of inflammation and OS mediators (Supporting Table 2). This analysis revealed significant relationships, suggesting a complex interplay among the range of variables included in the correlation study. In this concern, the main results obtained indicated that for the primary indicator of the inflammatory status (NO), some (poly)phenolic compounds exhibited a significant anti-inflammatory effect by inhibiting the elevation of this marker (trigalloyl hexoside, petunidin 3-*O*-*p*-coumaroylglucoside, quercetin diglucuronide, malvidin 3-*O*-*p*-coumaroylglucoside, peonidin 3-(6-*trans*-*p*-coumaroyl)-glucoside, malvidin 3-*O-*glucoside, and proanthocyanidin trimer (B-type)). Surprisingly, the correlation coefficients suggested a proinflammatory effect for catechin-gallocatechin, cyanidin 3-*O*-*p*-coumaroylglucoside, proanthocyanidin derivative, and ∑-viniferin (Supporting Table 2). To gain further insight into identifying the compounds responsible for anti-inflammatory activity, additional correlations were established with inflammatory mediators IL-6, IL-8, and TNFα. Again, this analysis identified the compounds capable of modulating the inflammatory and immunological mediators IL-6 (catechin-gallocatechin, cyanidin 3-*O*-*p*-coumaroylglucoside, proanthocyanidin derivative, ∑-viniferin, proanthocyanidin dimmer (B-type), *trans-*caftaric acid, *p*-coumaric acid, and quercetin glucuronide), IL-8 (trigalloyl hexoside, petunidin 3-*O*-*p*-coumaroylglucoside, quercetin diglucuronide, and peonidin 3-(6-*trans*-p-coumaroyl)-glucoside) and TNFα (proanthocyanidin trimer (B-type) and malvidin 3-*O*-glucoside) (Supporting Table 2).

The analysis of the correlation between individual phenolics released during digestion into the intestinal lumen with OS markers (ROS and GSH) suggested a significant positive relationship between the reduction of ROS and preservation of GSH for proanthocyanidin derivatives, trigalloyl glucoside, ∑-viniferin, and cyanidin 3-*O*-*p-*cumaroylglucoside (Supporting Table 2). Similarly, the enzymatic antioxidant activities of CAT, SOD, and GPX were significantly correlated with theasinesin, protocatechuic acid hexoside, stilbene tetramer, myricetin rhamnohexoside, and quercetin diglucuronide in the bioaccessible fraction.

Interestingly, despite the evidence concerning the capacity of winery byproducts’ (poly)phenols to polarize cells toward an anti-inflammatory phenotype (Figure 3), several compounds exhibited correlations compatible with a proinflammatory effect (Supporting Table 1). Contrary to common belief, it is reported that high doses (1%) of certain phenolic compounds such as epigallocatechin gallate *in vivo* displayed a proinflammatory effect, as evidenced by the increased production of several proinflammatory cytokines and the lipid inflammatory mediator (PGE_2_).^54^ It should therefore be kept in mind that dose is critical in determining the direction and magnitude of the effect of certain (poly)phenols on inflammatory response. In the same sense, it should define synergistic, additive, or antagonistic interactions between individual phenolics that are critical in establishing the final biological scope of bioaccessible fractions assessed.^55^ The sense of biological cooperation between individual (poly)phenols would depend on the absolute concentration and the relative proportion concerning the separate combinations considered. This fact is mirrored by the main results retrieved from the correlation analysis performed, which require further confirmation resorting to model systems including a restricted array of factors. This would be of special interest since understanding these interactions is crucial for optimizing the use of winery byproducts in developing functional foods or nutraceuticals with targeted anti-inflammatory properties. By carefully selecting and combining specific polyphenols (upon the application of the information provided by the correlation analyses and model systems), it would be suitable to enhance their therapeutic efficacy while minimizing potential adverse effects.^56^

In summary, the results retrieved from the present study support the biological interest of (poly)phenols of oenological residues (grape stems, grape pomace, and wine lees), which are released into the intestinal lumen during gastrointestinal digestion and are accessible for epithelial cells undergoing inflammatory processes. Accordingly, once uptaken by local cells, these compounds would prevent primary inflammation and secondary OS, thus contributing to digestive health, specifically in the frame of IBD. Interestingly, contrasting with previous descriptions in the literature showing a limited efficiency of controlling the molecular mechanisms responsible for inflammatory processes, the lower (poly)phenolic concentration featuring the bioaccessible fraction appeared more active and efficient in combating inflammation and secondary augment of ROS in cells. This fact suggests the potential application of intermediate materials obtained upon dehydration of enological residues in preventing and treating diverse IBD phenotypes, also contributing to restoring the redox balance disturbance featuring this pathological process. However, the main findings in the present work suggest that although bioactive (poly)phenols may act as powerful antioxidants in humans, the putative reactivity of the diverse individual compounds may give rise to diverse responses, which would be dependent on the specific inflammatory pathways or the dosage, among other factors. This situation limits unique cellular responses due to any individual phenolic compound but confirms the operability of phenolic pools in the intestinal lumen. According to this conclusion, the major outcomes might be interpreted as indicators of the promising applicability of winery byproducts toward bioactive and healthy ingredients. Nevertheless, additional modeling approaches complementing these *in vitro* studies to shed light on their capacity to prevent macrophage migration and polarize the differentiation and maturation of antigen-presenting cells into a tolerogenic phenotype are needed. Indeed, this additional research will provide information about the mechanisms of action and pathophysiological conditions that could take advantage of such biological activities.

## Supporting Information Available

Preparation of simulated gastric fluid (SGF) and simulated intestinal fluid (SIF) and Spearman correlation between the bioaccessible (poly)phenols of grape (*Vitis vinifera* L.) stems, grape pomace, and wine lees and markers and mediators of inflammation and oxidative stress (PDF)

## Abbreviations Used

ATCC: American Type Culture Collection
CAT: catalase
DCFDA: 2′,7′-dichlorofluorescein diacetate
ELISA: enzyme-linked immunosorbent assay
EMEM: Eagle’s minimum essential medium
FBS: fetal bovine serum
GPx: gluthatione peroxidase
IBD: inflammatory bowel disease
IL: interleukin
OS: oxidative stress
SOD: superoxide dismutase
TNF-α: toumor necrosis factor α

## References

[ref1] Gajendran M.; Loganathan P.; Catinella A. P.; Hashash J. G. A Comprehensive Review and Update on Crohn’s Disease. Disease-a-Month 2018, 64 (2), 20–57. 10.1016/j.disamonth.2017.07.001. 28826742

[ref2] Ben-Horin S.; Kopylov U.; Chowers Y. Optimizing Anti-TNF Treatments in Inflammatory Bowel Disease. Autoimmun. Rev. 2014, 13 (1), 24–30. 10.1016/j.autrev.2013.06.002. 23792214

[ref3] Caban M.; Lewandowska U. Polyphenols and the Potential Mechanisms of Their Therapeutic Benefits against Inflammatory Bowel Diseases. J. Funct. Foods 2022, 95, 105181 10.1016/j.jff.2022.105181.

[ref4] Costa-Pérez A.; Medina S.; Sánchez-Bravo P.; Domínguez-Perles R.; García-Viguera C. The (Poly)Phenolic Profile of Separate Winery By-Products Reveals Potential Antioxidant Synergies. Molecules 2023, 28 (5), 2081 10.3390/molecules28052081. 36903327 PMC10004379

[ref5] Qi Q.; Chu M.; Yu X.; Xie Y.; Li Y.; Du Y.; Liu X.; Zhang Z.; Shi J.; Yan N. Anthocyanins and Proanthocyanidins: Chemical Structures, Food Sources, Bioactivities, and Product Development. Food Rev. Int. 2022, 39 (7), 4581–4609. 10.1080/87559129.2022.2029479.

[ref6] Tamargo A.; Cueva C.; Silva M.; Molinero N.; Miralles B.; Bartolomé B.; Moreno-Arribas M. V. Gastrointestinal Co-Digestion of Wine Polyphenols with Glucose/Whey Proteins Affects Their Bioaccessibility and Impact on Colonic Microbiota. Food Res. Int. 2022, 155, 111010 10.1016/j.foodres.2022.111010. 35400421

[ref7] Lamothe S.; Guérette C.; Dion F.; Sabik H.; Britten M. Antioxidant Activity of Milk and Polyphenol-Rich Beverages during Simulated Gastrointestinal Digestion of Linseed Oil Emulsions. Food Res. Int. 2019, 122, 149–156. 10.1016/j.foodres.2019.03.068. 31229066

[ref8] Somaratne G.; Ferrua M. J.; Ye A.; Nau F.; Floury J.; Dupont D.; Singh J. Food Material Properties as Determining Factors in Nutrient Release during Human Gastric Digestion: A Review. Cri. Rev. Food Sci. Nutr. 2020, 60 (22), 3753–3769. 10.1080/10408398.2019.1707770. 31957483

[ref9] Domínguez-Perles R.; Moreno D. A.; Garcia-Viguera C. Waking Up from Four Decades’ Long Dream of Valorizing Agro-Food Byproducts: Toward Practical Applications of the Gained Knowledge. J. Agric. Food Chem. 2018, 66 (12), 3069–3073. 10.1021/acs.jafc.7b05848. 29526103

[ref10] Barros A.; Gironés-Vilaplana A.; Texeira A.; Baenas N.; Domínguez-Perles R. Grape Stems as a Source of Bioactive Compounds: Application towards Added-Value Commodities and Significance for Human Health. Phytochem. Rev. 2015, 14 (6), 921–931. 10.1007/s11101-015-9421-5.

[ref11] Machado N. F. L.; Domínguez-Perles R. Addressing Facts and Gaps in the Phenolics Chemistry of Winery By-Products. Molecules 2017, 22 (2), 286 10.3390/molecules22020286. 28216592 PMC6155862

[ref12] Directive 2008/98/EC of the European Parliament and of the Council of 19 November 2008 on Waste and Repealing Certain Directives; European Parliament: Brussels, July 5, 2008 http://data.europa.eu/eli/dir/2008/98/oj159.

[ref13] Sánchez-Bravo P.; Costa-Pérez A.; García-Viguera C.; Raúl Domínguez-Perles; Medina S. Prevention of Inflammation and Oxidative Stress by New Ingredients Based on High (Poly)Phenols Winery by-Products. J. Sci. Food Agric. Rep. 2025, 5, 1–10. 10.1002/jsf2.224.

[ref14] Minekus M.; Alminger M.; Alvito P.; Balance S.; Bohn T.; Bourlieu C.; Carrière F.; Boutrouo R.; Corredig M.; Dupont D.; Dufour C.; Egger L.; Golding M.; SKarakaya S.; BKirkhus B.; LeFeunteun S.; Lesmes U.; Macierzanka; Mackie A.; Marze S.; McClements D. J.; Ménard O.; Recio I.; Santos C. N.; Singh R. P.; Vegarud G. E.; Wickham M. S. J.; Weitschies W.; Brodkorb A. A Standardised Static *in Vitro* Digestion Method Suitable for Food - an International Consensus. Food Funct. 2014, 5 (6), 1113–1124. 10.1039/C3FO60702J. 24803111

[ref15] Brodkorb A.; Egger L.; Alminger M.; Alvito P.; Assunçao R.; Ballance S.; Balance S.; Bohn T.; Bourlieu-Lacanal C.; Boutrou R.; Carrière F.; Clemente A.; Corredig M.; Dupont D.; Dufour C.; Edwards C.; Edwards C.; Golding M.; Golding M.; Karakaya S.; Karakaya S.; Kirkus B.; Kirkhus B.; Le Feunteun S.; Le Feunteun S.; Lesmes U.; Lesmes U.; Macierzanka A.; Macierzanka A.; Mackie A. R.; Mackie A. R.; Martins C.; Martins C.; Marze S.; Marze S.; McClements D. J.; McClements D. J.; Ménad O.; Ménard O.; Minekus M.; Minekus M.; Portmann R.M.; Portmann R.; Santos C. N.; Santos C. N.; Souchon I.; Souchon I.; Singh R. P.; Singh R. P.; Vegarud G. E.; Vegarud G. E.; Wickham M. S. J.; Wickham M. S. J.; Witshies W.; Weitschies W.; Recio I. INFOGEST Static in Vitro Simulation of Gastrointestinal Food Digestion. Nat. Protoc. 2019, 14 (4), 991–1014. 10.1038/s41596-018-0119-1. 30886367

[ref16] Abellán Á.; Domínguez-Perles R.; García-Viguera C.; Moreno D. A. Evidence on the Bioaccessibility of Glucosinolates and Breakdown Products of Cruciferous Sprouts by Simulated In Vitro Gastrointestinal Digestion. Int. J. Mol. Sci. 2021, 22 (20), 11046 10.3390/ijms222011046. 34681712 PMC8539263

[ref17] Martínez Sánchez S.; Dominguez-Perles R.; Montoro S.; Gabaldón Hernández J. A.; Guy A.; Durand T.; Oger C.; Ferreres F.; Gil-Izquierdo A. Bioavailable Phytoprostanes and Phytofurans from Gracilaria Longissima Have Anti-Inflammatory Effects in Endothelial Cells. Food Funct. 2020, 11 (6), 5166–5178. 10.1039/D0FO00976H. 32432610

[ref18] Van De Walle J.; Hendrickx A.; Romier B.; Larondelle Y.; Schneider Y. J. Inflammatory Parameters in Caco-2 Cells: Effect of Stimuli Nature, Concentration, Combination and Cell Differentiation. Toxicol. in Vitro 2010, 24 (5), 1441–1449. 10.1016/j.tiv.2010.04.002. 20406675

[ref19] Domínguez-Perles R.; Guedes A.; Queiroz M.; Silva A. M.; Barros A. I. R. N. A. Oxidative Stress Prevention and Anti-Apoptosis Activity of Grape (*Vitis Vinifera* L.) Stems in Human Keratinocytes. Food Res. Int. 2016, 87, 92–102. 10.1016/j.foodres.2016.06.030. 29606253

[ref20] Queiroz M.; Oppolzer D.; Gouvinhas I.; Silva A. M.; Barros A. I. R. N. A.; Domínguez-Perles R. New Grape Stems’ Isolated Phenolic Compounds Modulate Reactive Oxygen Species, Glutathione, and Lipid Peroxidation *in Vitro*: Combined Formulations with Vitamins C and E. Fitoterapia 2017, 120, 146–157. 10.1016/j.fitote.2017.06.010. 28625733

[ref21] Du Plessis L.; Laubscher P.; Jooste J.; Du Plessis J.; Franken A.; Van Aarde N.; Eloff F. Flow Cytometric Analysis of the Oxidative Status in Human Peripheral Blood Mononuclear Cells of Workers Exposed to Welding Fumes. J. Occup. Environ. Hyg. 2010, 7 (6), 367–374. 10.1080/15459621003724108. 20397091

[ref22] Ma X.; Li Y.; Lv C.; Liu B.; Yuan C.; Huang W.; Luo Q.; Xiao Y.; Sun C.; Li T.; Zhang J. Modulation of Keap1-Nrf2-ARE Signaling Pathway by Oxyresveratrol, a Derivative of Resveratrol from Grape Skin. Food Biosci. 2022, 50, 102162 10.1016/j.fbio.2022.102162.

[ref23] Gómez-Zorita S.; Milton-Laskibar I.; Eseberri I.; Beaumont P.; Courtois A.; Krisa S.; Portillo M. P. Beneficial Effects of ε-Viniferin on Obesity and Related Health Alterations. Nutrients 2023, 15 (4), 928 10.3390/nu15040928. 36839286 PMC9963111

[ref24] Alvarez-Suarez J. M.; Giampieri F.; Tejera E.; Battino M. Malvidin: Advances in the Resources, Biosynthesis Pathway, Bioavailability, Bioactivity, and Pharmacology. Handb. Diet. Flavonoids 2023, 1–35. 10.1007/978-3-030-94753-8_57-1.

[ref25] Ovando-Martínez M.; Gámez-Meza N.; Molina-Domínguez C. C.; Hayano-Kanashiro C.; Medina-Juárez L. A. Simulated Gastrointestinal Digestion, Bioaccessibility and Antioxidant Capacity of Polyphenols from Red Chiltepin (*Capsicum Annuum* L. Var. Glabriusculum) Grown in Northwest Mexico. Plant Foods Hum. Nutr. 2018, 73 (2), 116–121. 10.1007/s11130-018-0669-y. 29700672

[ref26] Mosele J. I.; Macià A.; Romero M. P.; Motilva M. J. Stability and Metabolism of Arbutus Unedo Bioactive Compounds (Phenolics and Antioxidants) under *in Vitro* Digestion and Colonic Fermentation. Food Chem. 2016, 201, 120–130. 10.1016/j.foodchem.2016.01.076. 26868556

[ref27] Costa-Pérez A.; Moreno D. A.; Periago P. M.; García-Viguera C.; Domínguez-Perles R. A New Food Ingredient Rich in Bioaccessible (Poly)Phenols (and Glucosinolates) Obtained from Stabilized Broccoli Stalks. Foods 2022, 11 (12), 1734 10.3390/foods11121734. 35741932 PMC9222756

[ref28] Teixeira A.; Baenas N.; Dominguez-Perles R.; Barros A.; Rosa E.; Moreno D. A.; Garcia-Viguera C. Natural Bioactive Compounds from Winery By-Products as Health Promoters: A Review. Int. J. Mol. Sci. 2014, 15 (9), 15638–15678. 10.3390/ijms150915638. 25192288 PMC4200838

[ref29] Gil-Sánchez I.; Cueva C.; Tamargo A.; Quintela J. C.; de la Fuente E.; Walker A. W.; Moreno-Arribas M. V.; Bartolomé B. Application of the Dynamic Gastrointestinal Simulator (Simgi) to Assess the Impact of Probiotic Supplementation in the Metabolism of Grape Polyphenols. Food Res. Int. 2020, 129, 108790 10.1016/j.foodres.2019.108790. 32036893

[ref30] Renard C. M. G. C.; Watrelot A. A.; Le Bourvellec C. Interactions between Polyphenols and Polysaccharides: Mechanisms and Consequences in Food Processing and Digestion. Trends Food Sci. Technol. 2017, 60, 43–51. 10.1016/j.tifs.2016.10.022.

[ref31] Mihaylova D.; Desseva I.; Stoyanova M.; Petkova N.; Terzyiska M.; Lante A. Impact of In Vitro Gastrointestinal Digestion on the Bioaccessibility of Phytochemical Compounds from Eight Fruit Juices. Molecules 2021, 26, 1187 10.3390/molecules26041187. 33672156 PMC7927027

[ref32] Menchaca-Armenta M.; José Frutos M.; Ramírez-Wong B.; Valero-Cases E.; Muelas-Domingo R.; Quintero-Ramos A.; Isabel Torres-Chávez P.; Carbonell-Barrachina Á. A.; Irene Ledesma-Osuna A.; Nydia Campas-Baypoli O. Changes in Phytochemical Content, Bioaccesibility and Antioxidant Capacity of Corn Tortillas during Simulated *in Vitro* Gastrointestinal Digestion. Food Chem. 2023, 405, 134223 10.1016/j.foodchem.2022.134223. 36403465

[ref33] Deseva I.; Stoyanova M.; Petkova N.; Mihaylova D. Red Beetroot Juice Phytochemicals Bioaccessibility: An In Vitro Approach. Polym. J. Food Nutr. Sci. 2020, 70 (1), 45–53. 10.31883/pjfns/116590.

[ref34] Desseva I.; Mihaylova D. Influence of *in Vitro* Gastrointestinal Digestion on Phytochemicals in Pomegranate Juice. Food Sci. Technol. 2020, 40, 211–216. 10.1590/fst.07219.

[ref35] Lingua M. S.; Theumer M. G.; Kruzynski P.; Wunderlin D. A.; Baroni M. V. Bioaccessibility of Polyphenols and Antioxidant Properties of the White Grape by Simulated Digestion and Caco-2 Cell Assays: Comparative Study with Its Winemaking Product. Food Res. Int. 2019, 122, 496–505. 10.1016/j.foodres.2019.05.022. 31229105

[ref36] Luissint A. C.; Parkos C. A.; Nusrat A. Inflammation and the Intestinal Barrier: Leukocyte–Epithelial Cell Interactions, Cell Junction Remodeling, and Mucosal Repair. Gastroenterology 2016, 151 (4), 616 10.1053/j.gastro.2016.07.008. 27436072 PMC5317033

[ref37] Zhang J. M.; An J. Cytokines, Inflammation, and Pain. Int. Anesthesiol. Clin. 2007, 45 (2), 27–37. 10.1097/AIA.0b013e318034194e. 17426506 PMC2785020

[ref38] Peterson L. W.; Artis D. Intestinal Epithelial Cells: Regulators of Barrier Function and Immune Homeostasis. Nat. Rev. Immunol. 2014, 14 (3), 141–153. 10.1038/nri3608. 24566914

[ref39] Abreu M. T.; Palladino A. A.; Arnold E. T.; Kwon R. S.; McRoberts J. A. Modulation of Barrier Function during Fas-Mediated Apoptosis in Human Intestinal Epithelial Cells. Gastroenterology 2000, 119 (6), 1524–1536. 10.1053/gast.2000.20232. 11113074

[ref40] Schwingshackl L.; Schwedhelm C.; Galbete C.; Hoffmann G. Adherence to Mediterranean Diet and Risk of Cancer: An Updated Systematic Review and Meta-Analysis. Nutrients 2017, 9 (10), 1063 10.3390/nu9101063. 28954418 PMC5691680

[ref41] Huang F. C. The Interleukins Orchestrate Mucosal Immune Responses to Salmonella Infection in the Intestine. Cells 2021, 10 (12), 3492 10.3390/cells10123492. 34943999 PMC8700606

[ref42] Sharma J. N.; Al-Omran A.; Parvathy S. S. Role of Nitric Oxide in Inflammatory Diseases. Inflammopharmacology 2007, 15 (6), 252–259. 10.1007/s10787-007-0013-x. 18236016

[ref43] Wang X. M.; Hamza M.; Wu T. X.; Dionne R. A. Upregulation of IL-6, IL-8 and CCL2 Gene Expression after Acute Inflammation: Correlation to Clinical Pain. Pain 2009, 142 (3), 275–283. 10.1016/j.pain.2009.02.001. 19233564 PMC3513699

[ref44] Bickel M. The Role of Interleukin-8 in Inflammation and Mechanisms of Regulation. J. Periodontol. 1993, 64, 456–460. 8315568

[ref45] Andrews C.; McLean M. H.; Durum S. K. Cytokine Tuning of Intestinal Epithelial Function. Front. Immunol. 2018, 9, 368738 10.3389/fimmu.2018.01270. PMC5996247 29922293

[ref46] Christofoletti C. R.; Fernandes A. C. F.; Gandra R. L. P.; Martins I. M.; Gambero A.; Macedo G. A.; Macedo J. A. Wine Residues Extracts Modulating *in Vitro* Metabolic Syndrome. Food Biosci. 2022, 50, 101957 10.1016/j.fbio.2022.101957.

[ref47] Del Pino-García R.; Gerardi G.; Rivero-Pérez M. D.; González-SanJosé M. L.; García-Lomillo J.; Muñiz P. Wine Pomace Seasoning Attenuates Hyperglycaemia-Induced Endothelial Dysfunction and Oxidative Damage in Endothelial Cells. J. Funct. Foods 2016, 22, 431–445. 10.1016/j.jff.2016.02.001.

[ref48] Tekos F.; Skaperda Z.; Vardakas P.; Kyriazi D.; Maravelis G. C.; Poulas K.; Taitzoglou I. A.; Nepka C.; Kouretas D. Redox Biomarkers Assessment after Oral Administration of Wine Extract and Grape Stem Extract in Rats and Mice. Molecules 2023, 28 (4), 1574 10.3390/molecules28041574. 36838560 PMC9965357

[ref49] Gaucher C.; Boudier A.; Bonetti J.; Clarot I.; Leroy P.; Parent M. Glutathione: Antioxidant Properties Dedicated to Nanotechnologies. Antioxidants 2018, 7 (5), 62 10.3390/antiox7050062. 29702624 PMC5981248

[ref50] Jung K. A.; Kwak M. K. The Nrf2 System as a Potential Target for the Development of Indirect Antioxidants. Molecules 2010, 15 (10), 7266–7291. 10.3390/molecules15107266. 20966874 PMC6259123

[ref51] Hussain T.; Tan B.; Yin Y.; Blachier F.; Tossou M. C. B.; Rahu N. Oxidative Stress and Inflammation: What Polyphenols Can Do for Us? Oxid. Med. Cell Longevity 2016 10.1155/2016/7432797. PMC5055983 27738491

[ref52] Ramos-Escudero F.; Muñoz A. M.; Alvarado-Ortíz C.; Alvarado Á.; Yáñez J. A. Purple Corn (*Zea Mays* L.) Phenolic Compounds Profile and Its Assessment as an Agent against Oxidative Stress in Isolated Mouse Organs. J. Med. Food 2012, 15 (2), 206–215. 10.1089/jmf.2010.0342. 22082063 PMC3264953

[ref53] Margină D.; Ungurianu A.; Purdel C.; Niţulescu G. M.; Tsoukalas D.; Sarandi E.; Thanasoula M.; Burykina T. I.; Tekos F.; Buha A.; Nikitovic D.; Kouretas D.; Tsatsakis A. M. Analysis of the Intricate Effects of Polyunsaturated Fatty Acids and Polyphenols on Inflammatory Pathways in Health and Disease. Food Chem. Toxicol. 2020, 143, 111558 10.1016/j.fct.2020.111558. 32640331 PMC7335494

[ref54] Pae M.; Ren Z.; Meydani M.; Shang F.; Smith D.; Meydani S. N.; Wu D. Dietary Supplementation with High Dose of Epigallocatechin-3-Gallate Promotes Inflammatory Response in Mice. J. Nutr. Biochem. 2012, 23 (6), 526–531. 10.1016/j.jnutbio.2011.02.006. 21684134

[ref55] Ighodaro O. M.; Akinloye O. A. First Line Defence Antioxidants-Superoxide Dismutase (SOD), Catalase (CAT) and Glutathione Peroxidase (GPX): Their Fundamental Role in the Entire Antioxidant Defence Grid. Alexandria J. Med. 2018, 54 (4), 287–293. 10.1016/j.ajme.2017.09.001.

[ref56] Vicol C.; Duca G. Synergistic, Additive and Antagonistic Interactions of Some Phenolic Compounds and Organic Acids Found in Grapes. Acta Chim. Slov. 2023, 70 (4), 588–600. 10.17344/acsi.2023.8214. 38124647

