# Supplementary material for: Bioaccessible (Poly)phenols of Winery Byproducts Modulate Pathogenic Mediators of Intestinal Bowel Disease: In Vitro Evidence
Source: J Agric Food Chem. 2025 Apr 23;73(18):11007–18. doi: 10.1021/acs.jafc.5c00916 (PMC12063181; doi:10.1021/acs.jafc.5c00916)
Supplement: Supplementary file 1 — jf5c00916_si_001.pdf [file jf5c00916_si_001.pdf]

Supporting Information

Bioaccessible (poly)phenols of winery by-products modulate pathogenic mediators of intestinal bowel disease: *in vitro* evidence

Vicente Agulló[a], Cristina García-Viguera[b], Sonia Medina[b,*], Raúl Domínguez-Perles[b]

[a] *Departamento de Tecnología Agroalimentaria, EPSO, Universidad Miguel Hernández, Carretera Beniel km 3.2, 03312 Orihuela, Alicante, Spain.*

[b] *Laboratorio de Fitoquímica y Alimentos Saludables (LabFAS), CEBAS, CSIC, Campus Universitario de Espinardo, Edificio 25, 30100 Murcia, Spain.*

Supplemental Table 1. Preparation of simulated gastric fluid (SGF) and simulated intestinal fluid (SIF).

Simulated fluids	Constituent [Z] (mmol/L)					
	KCl	KH_2PO_4	$NaHCO_3$	NaCl	$MgCl_2(H_2O)_6$	$(NH_4)_2CO_3$
Simulated Gastric Fluid (SGF; pH 3)	6.90	0.90	25.00	47.20	0.10	0.50
Simulated Intestinal Fuid (SIF; pH 8)	6.80	0.80	85.00	38.40	0.13	---

[Z] KCl, potassium chloride; KH_2PO_4, potassium phosphate monobasic; $NaHCO_3$, sodium bicarbonate; NaCl, sodium chloride; $MgCl_2(H_2O)_6$, magnesium chloride hexahydrate; $(NH_4)_2CO_3$, ammonium carbonate.

Supplementary Table 2. Spearman correlation (r^2) between the bioaccessible (poly)phenols of grape (*Vitis vinifera* L.) stems, grape pomace, and wine lees and markers and mediators of inflammation (nitric oxide, interleukin (IL)-6, IL-8, and TNF-α) and oxidative stress (reactive oxygen species (ROS), glutathione (GSH), catalase (CAT), super oxide dismutase (SOD), and Glutathione peroxidase (GPx)).

Phenolic compound	Inflammatory marker				Oxidative stress markers and mediators				
	NO	IL-6	IL-8	TNF-α	ROS	GSH	CAT	SOD	GPx
Proanthocyanidin dimmer (B-type)	N.s.	**-0.480***	N.s.	N.s.	N.s.	**0.544***	-0.674*	0.753**	-0.793**
Proanthocyanidin trimer (B-type)	**-0.544***	0.540*	N.s.	**-0.413***	0.962**	-0.544*	-0.595*	N.s.	N.s.
Catechin-gallocatechin	0.890**	**-0.900****	0.630**	N.s.	0.544*	-0.962**	N.s.	N.s.	N.s.
Theanesin	N.s.	0.480*	N.s.	N.s.	N.s.	-0.544*	**0.674***	**-0.753****	**0.793****
Proanthocyanidin derivative	0.825**	**-0.828****	0.621**	N.s.	**-0.544***	**0.962****	N.s.	N.s.	N.s.
Trigalloyl hexoside	**-0.890****	0.900**	**-0.630****	N.s.	**-0.866****	**0.866****	N.s.	N.s.	N.s.
Protocatechuic acid hexoside	N.s.	0.480*	N.s.	N.s.	**-0.893****	-0.544*	**0.674****	**-0.753****	**0.793****
Trans-caftaric acid	N.s.	**-0.480***	N.s.	N.s.	N.s.	**0.544***	-0.674**	0.753**	-0.793**
Coumaric acid	N.s.	**-0.480***	N.s.	N.s.	N.s.	**0.544***	-0.674**	0.753**	-0.793**
Σ-viniferin	0.544*	**-0.540***	N.s.	0.413*	**-0.962****	**0.544***	**0.595****	N.s.	N.s.
Stilbenoid tetramer (hopeaphenol)	N.s.	0.480*	N.s.	N.s.	N.s.	-0.544*	**0.674****	**-0.753****	**0.793****
Myricetin rhamnohexoside	N.s.	0.480*	N.s.	N.s.	N.s.	-0.544*	**0.674****	**-0.753****	**0.793****
Quercetin glucuronide	N.s.	**-0.480***	N.s.	N.s.	N.s.	**0.544***	-0.674**	0.753**	-0.793**
Quercetin diglucuronide	**-0.753****	0.767**	**-0.500***	N.s.	N.s.	-0.837**	N.s.	**-0.628****	**0.662****
Malvidin 3-*O*-glucoside	**-0.544***	0.540*	N.s.	**-0.413***	0.962**	-0.544*	-0.595*	N.s.	N.s.
Cyanidin 3-*O*-*p*-coumaroylglucoside	0.890**	**-0.900****	0.630**	N.s.	**-0.544***	**0.962****	N.s.	N.s.	N.s.
Petunidin 3-*O*-*p*-coumaroylglucoside	**-0.890****	0.900**	**-0.630****	N.s.	0.544*	-0.962**	N.s.	N.s.	N.s.
Malvidin 3-*O*-*p*-coumaroylglucoside	**-0.544***	0.540*	N.s.	0.720**	0.962**	-0.544*	-0.595*	N.s.	N.s.
Peonidin 3-(6-*trans*-*p*-coumaroyl)-glucoside	**-0.544***	0.540*	**-0.450***	0.720**	0.962**	-0.544*	-0.595*	N.s.	N.s.

N.s., not significant at $p < 0.05$; * significant at $p < 0.05$; and ** significant at $p < 0.01$.
